# Enzyme Activity and Physiochemical Properties of Flour after Supercritical Carbon Dioxide Processing

**DOI:** 10.3390/foods11131826

**Published:** 2022-06-21

**Authors:** Maja Leitgeb, Željko Knez, Gordana Hojnik Podrepšek

**Affiliations:** 1 Faculty of Chemistry and Chemical Engineering, University of Maribor, Smetanova ulica 17, 2000 Maribor, Slovenia; zeljko.knez@um.si (Ž.K.); gordana.hojnik@um.si (G.H.P.); 2 Faculty of Medicine, University of Maribor, Taborska ulica 8, 2000 Maribor, Slovenia

**Keywords:** enzymes, enzyme activity, proteins, supercritical fluids, wheat flour

## Abstract

The objectives of this study were to inactivate the enzymes α-amylase, lipase, protease, and peroxidase in flour with supercritical carbon dioxide (scCO_2_), and to optimize the enzymatic treatment conditions. Enzyme inactivation is important, due to the undesirability of certain flour enzymes that cause adverse reactions during storage as unpleasant rancidity of flour, and, at the same time, reduce the shelf life of flour. Therefore, crude enzymes and flour were initially exposed to scCO_2_ to determine the effect on specific enzyme activity under appropriate conditions. The activity of the unwanted enzymes lipase and peroxidase decreased under optimal process conditions of scCO_2_ exposure, lipase by 30%, and peroxidase by 12%, respectively. It was discovered that the inactivation of enzymes in wheat flour occurred, where, at the same time, this sustainable method allows the regulation of enzyme activity in the baking process. Afterwards, the effect of scCO_2_ on the physicochemical properties of flour, morphological changes on starch granules, and content of total lipids was studied. In scCO_2_-treated white wheat flour, the fat content decreased by 46.15 ± 0.5%, the grain structure was not damaged, and the bread as the final product had a lower specific surface volume. Therefore, this could be a promising technology for flour pretreatment, potentially impacting the prolonging of its shelf-life.

## 1. Introduction

The numerous enzymes present in the bran and germ fractions of a wheat kernel initiate many chemical changes that affect the compositional and functional properties of whole wheat flour. In general, wheat flour is composed mainly of macronutrients such as starch, water, proteins, and non-starch polysaccharides, such as lipids and ashes [1], and also contains several technologically important enzymes, mainly amylases, proteases, lipoxygenase, polyphenol oxidase, and peroxidase. In addition, there are the three most important groups of enzymes with regard to the process of baking: Enzymes that hydrolyse carbohydrates, such as cellulase, amylases, and pentosanases, enzymes that hydrolyse proteins, such as proteases, and enzymes that affect fats and oils, mainly lipase and lipoxygenases. Although the enzymes are inactive during the storage of grain and flour, they become active when water is added, and, thus, play a significant role in determining the functional attributes of flour [2].

One of the challenges faced by the food industry is the stability of whole wheat flour during storage [3]. The development of hydrolytic and oxidative rancidity during storage decreases the sensory acceptability, as well as the compositional and functional properties of flour [4,5]. For these reasons, maintaining the quality of whole wheat flour to be used in product development and formulations is a challenge to milling companies and the food industry. The accumulation of free fatty acids in flour during storage is related positively to the initial lipase activity of flour. Reducing/inactivating lipase activity would be one of the methods to reduce the accumulation of free fatty acids. Another class of enzymes, peroxidases which are oxidoreductase enzymes, use hydrogen peroxide or organic hydroperoxides as oxidants. Peroxidase also plays a significant role in carotenoid bleaching during dough mixing, and may be responsible for the undesirable browning of flour [6].

Previous studies have used different thermal processing methods, including steaming, microwave heating, and passing through infrared and gamma radiation, to decrease enzyme activity [7,8,9]. The disadvantage of these methods is the use of high temperatures, which, besides high energy consumption, also affect the quality of the food product. Conventional heat treatment applications cause nutritional loss and undesired changes in sensorial qualities. Moreover, an excessively high concentration of protease in wheat flour can result in a total breakdown of the gluten protein structure, and a small amount of enzyme α-amylase is necessary to break down starch, which then provides an adequate supply of fermentable sugars. This is often supplied by the addition of malt or commercial preparations of fungal or bacterial α-amylase [10]. However, if an excess enzyme is present, starch is broken down to dextrins and simple sugars, resulting in a sticky, hard-to-handle dough that produces bread with a wet, sticky crumb [11].

This paper is focused on the enzyme activities and compositional properties of different flour types for different applications. Optimization of the protein extraction process was performed, in order to describe the differences in the protein concentrations in various flour types. Furthermore, another aim of this work was to observe the effects of scCO_2_ treatment on the compositional and functional properties of whole wheat flour, as CO_2_ is a thermodynamically stable molecule with low reactivity [12]. In principle, the technology of scCO_2_ treatment, as a green, waterless, more energy-efficient, cleaner, and safer production method, has attracted considerable interest in different industries in recent years [13]. ScCO_2_ is used commonly, and has become the solvent of choice for the food, cosmetic and pharmaceutical industries as a result of its non-toxicity and moderate critical properties (critical pressure: 7.38 MPa, critical temperature: 304.35 K) [14]. It is well known that, in scCO_2_, the most widely used supercritical fluid, enzyme activity may be tuned with different pressure/temperature/time combinations, and, at certain conditions, it may be reduced [15,16]. This could be attributed to the formation of carbonic acid in the presence of water and carbamates by reaction with amine groups on the enzyme [17]. Under certain pressure and temperature conditions, a change in the protein structure can happen with an increase in the β-sheet structure that leads to the burial of the catalytic center [18]. Subsequently, scCO_2_ treatment of flour may reduce enzymatic activities efficiently without thermal treatment, ensuring the same quality of flour after this process is finished. Due to the undesirable effects of some enzymes, a series of experiments were performed to achieve the inactivation of these enzymes in wheat flour.

Certainly, an important parameter is scCO_2_’s solubility, which is needed for understanding the fermentation of bread dough modeling. Fan et al. researched CO_2_ solubility in aqueous dough during baking, and found that CO_2_ solubility is responsible for the early oven rise of the dough, since vapor makes a significant contribution to the later expansion [19]. Therefore, the solubilities of CO_2_ were also measured in different flour types.

In addition white wheat flour was exposed to scCO_2_, with the aim to inactivate the enzymes in the flour. Thus, after exposure of the flour to scCO_2_, the proteins were extracted, and the residual activities of the enzymes α-amylase, lipase, protease, and peroxidase were determined in the supernatant. Moreover, a comparison was conducted between the enzyme activity in unexposed flour to the residual enzyme activity in scCO_2_ treated flour and the activities of free enzymes treated in scCO_2_. To understand the mechanism of enzyme inactivation using scCO_2_ it is essential to know the interactions between biological molecules like amino acids, proteins, and CO_2_. Amino acids are the structural constituents of the proteins, containing amine (NH_2_) and carboxyl (COOH) functional groups with a side chain (R group—aliphatic, acyclic, aromatic) specific to each amino acid. Moreover, an aqueous solution of amino acids and amino acid salts is advantageous in capturing CO_2_ where dipole-quadrupole interactions become significant in the investigation of such complicated thermodynamic systems (water + amino acid + carbon dioxide) [20]. The fundamental chemical modifications in the amino acids exposed to SCCO_2_ are oxidation, sulfonation, hydroxylation, ring-opening, and amidation [21], wherein the inactivation of enzymes is mostly attributable to the type of amino acid in the active site [22]. The knowledge gained from this study may also help promote the application of a sustainable approach of using scCO_2_ in the food industry and food science.

## 2. Materials and Methods

White wheat flour type 500, wheat flour type 850, rye flour type 1250, wholegrain rye flour, graham flour, and wholegrain spelt flour were kindly supplied by the bakery Hlebček, d.o.o. (Pragersko, Slovenia). Experiments were conducted using carbon dioxide (99.5% purity) produced by Messer, Ruše. Ethanol (96%), phosphoric acid (≥85%), sodium chloride (≥99.5%), Coomassie-Brilliant Blue G250 (1.15444.0025) and acetonitrile (99.9%) were supplied from Merck, while the bovine serum albumin (≥96%), sodium acetate (≥99.0%), acetic acid (GR for analysis), p-nitrophenyl butyrate (≥98%) were supplied from Sigma. The enzymes α-amylase (~30 U/mg) from *Aspergillus oryzae* and protease (≥0.6 U/mg) from *Aspergillus saitoi* were obtained from Sigma, while the lipase (~200 U/g) from *Aspergillus niger* was obtained from BioChemics. Peroxidase from horseradish (232-668-6) was purchased from BBI Enzymes (UK). The petroleum ether (32247) used for lipid extraction was supplied by Honeywell, Riedel-de Haen. All other chemicals used in the laboratory were of analytical grade.

### 2.1. Protein Extraction Process

The extraction of proteins was performed in 0.1 M acetate buffer pH 5.3 with 5 g of wheat flour in an Erlenmeyer flask, followed by incubation at room temperature for a specific time with horizontal shaking at 300 rpm. Afterwards, centrifugation at 8000 rpm was performed for five minutes. To optimize the extraction process, three different combinations of centrifugation and filtration were performed, to obtain the highest value of protein concentration. The first sample was, thus, filtered through a filter with a pore size of 0.45 µm. For the second sample, additional centrifugation was performed under the same conditions (8000 rpm, five minutes). In the third sample, the supernatant was decanted after the first centrifugation. For all three combinations, the supernatant was separated and considered as a crude protein extract. The protein concentration was determined in the supernatant according to the Bradford method [23]. Each sample was assayed twice, and three individual absorbance measurements per extracted sample were recorded within an experimental error of about 0.03%.

### 2.2. scCO_2_ Medium Treatment

An application of scCO_2_ technology was implemented to determine the effects of scCO_2_ on enzymes’ activities. First, the native enzymes were exposed to scCO_2_ under different conditions in a high-pressure batch reactor, and then white wheat flour was exposed to the scCO_2_ medium under the same conditions. A high-pressure batch reactor with a volume of 60 mL with a CO_2_ gas supply was used in the experimental research. When the temperature in the high-pressure batch reactor reached 35 °C, the pressure was raised to the desired value of 200 and 300 bar with cooled CO_2_. Afterwards, the system was maintained at a constant temperature and pressure for a preestablished exposure time. After the scCO_2_ medium treatment, the reactor was depressurized rapidly (0.37 ± 0.08 bar/min). Later, enzymes were extracted from the scCO_2_ treated wheat flour, where the supernatant was used to determine the enzyme activity and protein concentration. Residual enzyme activities in the treated wheat flour were correlated to the enzyme activities in untreated wheat flour, taken as 100%. The flour samples were subjected to the same conditions of scCO_2_ treatment for three times to ensure repeatability, whereby the percentage error was ±2.0%.

### 2.3. Flour Physicochemical Analyses

#### 2.3.1. Moisture Content

The moisture was determined from the weight loss of a sample dried by heating to 130 °C. Moisture determination in the flour samples was repeated three times, to obtain reliable results within an experimental error of about 0.5%.

#### 2.3.2. Particle Size Analysis

Previous studies have shown that the particle size distribution of flours from all wheat types could be measured more precisely by laser diffraction than by sieve analysis [24]. Therefore, the particle size distribution of flour samples was measured by a laser diffraction particle size analyzer (Analysette 22, Fritsch, Schlieren, Switzerland), using the dry analysis method within the selected size range from 0.3 to 300 µm. The sample was placed into the sample hopper manually and then aspirated into the apparatus. The particles cross the laser beam, and the particle size distribution was measured in this way. The distribution was reported both as a Gaussian curve and arithmetic mean (µm). Subsequently, the flour particle size distributions were determined in triplicate measurements with an experimental error of 0.5%.

#### 2.3.3. Environmental Scanning Electron Microscope

A Quanta FEI 200 3D, Oregon, United States microscope was used in environmental mode to observe the morphological changes in the starch granules in the flour that occurred during the scCO_2_ high-pressure treatment ESEM. The pressure chamber was 60 Pa, and the accelerating voltage for imaging was 10 kV.

#### 2.3.4. Determination of CO_2_ Solubility in Flour with Magnetic Suspension Balance (MSB)

**Figure 1:**
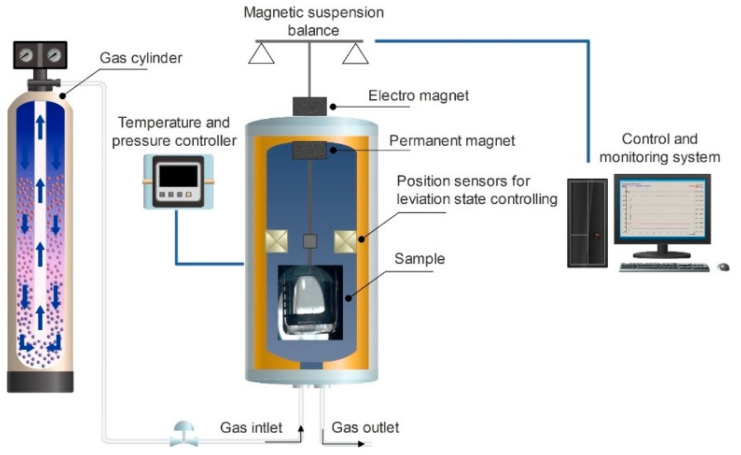
Schematic presentation of the magnetic suspension balance.

The phase equilibria behavior was studied by determination of the CO_2_’s solubility and the volumetric expansion of flour saturated with CO_2_. The solubility of CO_2_ is the total amount of carbonate species that can be dissolved in the solution. The solubilities of CO_2_ in different flour types were measured by a gravimetric method involving magnetic suspension balance (MSB, RUBOTHERM, Berlin, Germany) at pressures ranging from 1 to 350 bar (Figure 1). The applied MSB is designed for a maximum operating pressure of 350 bar and an operating temperature of 562 K. The temperature of the cell was kept constant using a Lauda P5 thermostat (Lauda, Königshofen, Germany). The measuring cell of the MSB has a sapphire window, which allows observation of the sample and estimation of volume modifications during the sorption measurements. The software MessPro (SL-2500-00216, Ver.11.05) recorded the conditions (mass, temperature, pressure). The duration of the measurement at each pressure was approximately 120 min, as equilibrium was reached after this time. To define the volume of the sample, a photo of the sample was taken with a digital camera and analyzed using ImageJ 1.52 software (Freeware).

#### 2.3.5. Fourier Transform Infrared Spectroscopy

Fourier transform infrared spectroscopy (FTIR, IRAffinity-1s, Shimadzu, Slovenia) using an ATR accessory was performed for the untreated flour and flour samples after scCO_2_ medium treatment with rapid and slow expansion. FTIR analysis was conducted to observe the changes in functional groups qualitatively, and further evaluate the modifications in the components of starch granules due to the impact of the scCO_2_ process.

#### 2.3.6. Determination of the Total Lipids in the Flour

Lipids are a group of substances that are soluble in organic solvents [25]. In general, Soxhlet extraction is one of the methods used most commonly for the determination of total lipids in dried foods. Therefore, a Soxhlet apparatus was used to extract the lipids from the untreated and scCO_2_ treated flour samples into petroleum ether. A total of 10 g of flour sample was used to extract the lipids with 180 mL of petroleum ether, boiling at 60 °C for 5 h [26]. In the Soxhlet extraction, the solvent was accumulated in the extraction chamber (the flour sample was held in a filter paper thimble) for 5–10 min and then returned to the boiling flask. This method provides a soaking effect for the sample. The solvent in the round bottom flask with the dissolved lipids was removed after a certain period of time, and the lipids were isolated by evaporating the organic solvent using a rotavapor apparatus at 40 °C, which is used for the recovery of lipids from the solution to near dryness. The mass of the extracted lipids obtained was determined by calculating the extraction yield.

Moreover, the percent of lipid content in the sample was calculated using Equation (1),

(1) $$\%\;Lipids=\frac{m_{extract}}{m_{flour\;sample}}*100$$

where *m_extract_* is the mass of lipids weighed after evaporating the petroleum ether, and *m_flour_* sample is the mass of flour used for lipid extraction.

### 2.4. Monitoring of Enzyme Activity

The activity of a specific enzyme was determined based on enzymatic activity assays using a UV-spectrophotometer (Varian, Cary 50 Probe, Agilent Technologies, Santa Clara, CA, USA) at different wavelengths.

The α-amylase activity was determined based on the increase in the amount of glucose in the solution by using the DNS reagent (3,5-dinitrosalicylic acid). The absorbance was measured with a UV-Vis spectrophotometer at a wavelength of 540 nm.

The activity of lipase was determined based on P-nitrophenol (pNP) reaction, which was monitored at 400 nm for 5 min. One unit of lipase activity released 1 nanomole of p-nitrophenol per minute at pH 7.2 and 37 °C using p-nitrophenyl butyrate (PNPB) as the substrate.

The protease activity was measured using casein as a substrate. The procedure for the determination of the protease activity was described previously in research [27].

The peroxidase activity was measured using phenol, 4-aminoantipyrine (4-AAP), and hydrogen peroxide as substrates. The method for peroxidase activity determination and a detailed calculation procedure of peroxidase activity has been published in our previous research [28].

The residual activities of different enzymes were calculated using the following Equation (2).

(2) $$residual\;activity\;\left(\%\right)=\frac{\;enzyme\;activity\;after\;scCO_{2\;}treatment\;}{enzyme\;activity\;before\;scCO_{2}\;treatment}*100$$

All experiments were carried out in triplicate, and the mean values express the average ± standard deviation.

#### Statistical Analysis

R version 4.1.1 and Rstudio, version 1.4.1717 were used to perform the statistical analysis and evaluate the differences between the residual enzyme activity within enzyme types in native form and in scCO_2_-treated flour after the performance of a two-way analysis of variance (ANOVA). The data were distributed normally and were presented with mean and standard deviation. A post hoc Tuckey honest significant difference test was performed to define the inactivation of enzymes in different groups.

### 2.5. Baking Test

The baking test was developed to establish the suitability of the flour for making bread, and to determine the difference between untreated and scCO_2_-treated white wheat flour in the bread making process. A bread-making machine, an SBB 850 F2, 850 W (SilverCrest, Lidl, Slovenia) was used for dough baking, according to the recipe from the bakery Hlebček d.o.o, Pragersko, Slovenia. The dough was prepared from 540 g of white wheat flour with 1.8% yeast, 2% salt, 58% water, and 2.5% of professional dough improver, which contains wheat flour, wheat gluten, sugar, wheat malt flour, flour treatment agent (ascorbic acid) and enzymes (α-amylase, hemicellulase). In general, bakeries add dough improver to improve the baking functionality in their bread production. For this purpose, a dough improver was added in the same quantity in baking bread with both types of flour, untreated and scCO_2_-treated white wheat flour. The ingredients were first preheated for 10 min, and the dough was mixed for 12 min and fermented for 20 min, whereas stirring and fermenting were then repeated for 7 min and 35 min. The bread loaf was baked for 60 min at 220 °C. The dough baking test was repeated with untreated white wheat flour and scCO_2_-treated white wheat flour under the same conditions and bread-making program. The loaves of bread were weighed after cooling, and their volume (cm^3^) was determined by the water displacement method. The specific volume (cm^3^/g) was calculated as loaf volume/bread weight. Values obtained were the mean of three replicates. A sensory analysis was carried out, involving evaluation of the sensory attributes of appearance, aroma, taste, and texture. Ten non-trained testers (8 females and 2 males, ranging in age between 26 and 58) made a hedonic evaluation of the acceptability of bread baked from untreated and scCO_2_-treated white wheat flour. In the test, participants were asked about their preferences concerning the differences in the experimental bread. The average height of the baked bread was measured, and determined using the ImageJ program.

## 3. Results

### 3.1. Optimization of the Flour Protein Extraction Process and Determination of the Protein Concentration

The conceptual design of the entire study started with the optimization of the protein extraction process and determination of the protein concentration, which were carried out with regard to six different flour types: Wheat flour type 500, wheat flour type 850, rye flour type 1250, wholegrain rye flour, graham flour, and wholegrain spelt flour. Since white wheat flour is the most commonly used, we decided to perform further experiments on this type of flour. Optimization of the protein extraction process was carried out to ensure the proper determination of the concentration of proteins. Optimal shaking time (30, 60 and 90 min) and a combination of the centrifugation and filtration procedures of the sample were modified experimentally using three different protocols, where, in the first procedure, the supernatant was obtained by centrifugation at 8000 rpm for 5 min followed by additional filtration through a 0.45 µm pore filter. In the second procedure, double centrifugation at 8000 rpm for 5 min was performed to obtain clear supernatant, and in the third procedure, single centrifugation at 8000 rpm for 5 min was performed to obtain clear supernatant. After the extraction of proteins from the wheat flour, protein concentration was determined by the Bradford method using a UV-Vis spectrophotometer at 595 nm.

The highest protein concentration was obtained after 90 min of shaking and single centrifugation in the Graham flour [29], followed by wheat flour type 850 and wholegrain spelt flour with a protein concentration of 4.9 mg/mL, 4.6 mg/mL, and 4.1 mg/mL. A lower protein concentration was detected in wheat flour type 500 with 3.1 mg/mL, and in wholegrain rye flour with 2.0 mg/mL. Regardless, the protein concentration was the lowest in rye flour type 1250 with a protein concentration of 1.5 mg/mL. Therefore, the extraction procedure of 90 min and single centrifugation, which ensured the highest protein concentration, was used in the further experiments.

The protein content of wheat varies widely, depending on the type or class of wheat, growing conditions, and fertilizer inputs, particularly nitrogen, whereas wheat flour proteins are related directly to bread volume. Therefore, protein quantity and quality have received considerable attention in bread-making processes. The behavior of the dough in the bread-making process depends primarily on the properties of the flour and its composition, resulting in the dough being able to retain gas bubbles during fermentation and baking, and leading to baked products with a high loaf volume and more regular crumb structure.

### 3.2. Flour Specification

Flour composition and quality are of vital importance in flour milling. The moisture content of wheat is important, and should not exceed 15%, being low enough to prevent spoilage. Table 1 presents the moisture content, fat value and protein value for the different flour types, which were used in our experiments. The moisture content of the flour is very important regarding its shelf life and storage stability. A moisture content over 14% affects the storage quality of flour negatively, due to mold growth, increase in microbial content, and infestation by insects. The deterioration in baking quality is also lower at a lower moisture content, which can be credited to the retarded respiration and activities of the microorganisms. In addition, fats affect the structural and mechanical properties of the dough and the texture of the products. If the fat content is high, not much water is needed to obtain the desired consistency, limiting gluten formation, swelling, and starch gelatinization. However, this correlation mainly depends on the type of flour [30]. Removing fat from the flour exposes the water binding sites on the side chain groups of protein units previously blocked in a lipophilic environment, thereby increasing water absorption capacity values in defatted flours [31]. On the other hand, defatted flour may contribute to designing a healthy human diet.

### 3.3. Effect of the scCO_2_ Medium on Enzyme Activity

When enzymes are exposed to scCO_2_, a different effect on their stability and activity may be observed, depending on the amount of water present, pressure and temperature of the treatment. Certainly, the type of enzyme is also crucial, as there are different mechanisms of enzyme inactivation in scCO_2_. The mechanism of enzyme inactivation in high-pressure treatments refers to the disruption of interactions that affect primarily the tertiary and quaternary enzyme structures, and result in the loss of the catalytic enzyme function. The high pressure causes protein denaturation, which occurs due to the penetration of water into the protein structure. The penetration of water into the protein matrix leads to conformational transitions that cause its unfolding [32]. Another possible effect of the enzyme’s inactivation in scCO_2_ treatment is the pH value. In principle, during the supercritical treatment, CO_2_ forms carbonic acid (H_2_CO_3_), which dissociates into bicarbonate (HCO^3−^), carbonate (CO_3_^2−^), and hydrogen (H^+^) ions, thereby reducing the pH inside the high-pressure reactor [33]. Yoshimura et al. reported that pH was lowered during scCO_2_ treatment, and caused the inactivation of α-amylase activity. Therefore, it was considered that α-amylase was inactivated predominantly by the pH-lowering effect of the microbubbles of scCO_2_ [34]. Furthermore, some amino acid residues can change their charge status as a function of pH. The change in amino acid residues’ charge in the active center of the enzyme leads to enzyme inactivation [35].

Based on these premises, the activities of the pure enzymes α-amylase, lipase, protease, and peroxidase were determined initially before and after the exposure to a scCO_2_ medium under certain conditions, in order to determine the suitability of the scCO_2_ method for the inactivation of enzymes.

**Figure 2:**
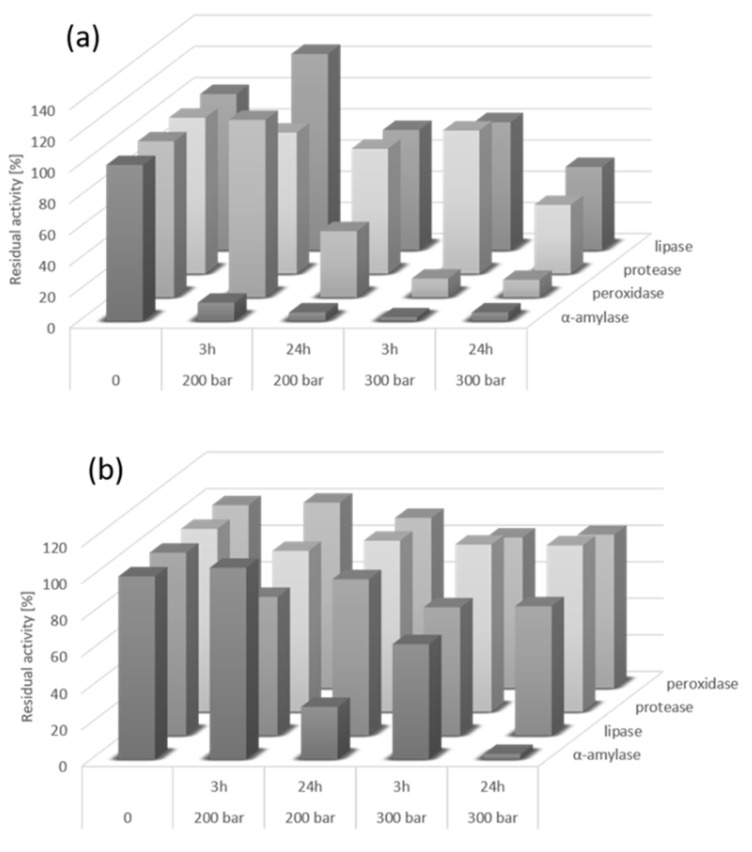
Determination of native enzymes’ (**a**) and wheat flour enzymes’ (**b**) activity after treatment at different scCO_2_ conditions (35 °C). The value of the untreated enzyme was set to 100%.

Figure 2a,b show the residual activities of the native enzymes α-amylase, lipase, peroxidase, and protease, and the same enzymes in wheat flour after scCO_2_ treatment under different conditions. The results in Figure 2a demonstrated that the native enzyme α-amylase is unstable, since its activity declined rapidly after three hours and 200 bar of scCO_2_ exposure. At 300 bar the decrease in its activity was even more significant. Similarly, a decrease in native peroxidase activity was observed to 12% after three hours of treatment at 300 bar.

In our study, the activities of lipase and protease decreased only after a longer exposure time of 24 h, as shown in Figure 2a. These two enzymes are well known as very stable in scCO_2_, and are very often used as biocatalysts in this medium. In this regard, Bauer et al. performed a treatment of esterase from porcine liver and lipase from *Candida rugosa* with scCO_2_ at different pressures with different amounts of water, at a temperature used commonly in biocatalysis with hydrolases. Lipase from *C. rugosa* was treated at 300 bar and 40 °C for 22 h, where the residual activity of the lipase was 91.6%. Moreover, the esterase from the porcine liver was treated at 200 bar and 65 °C for 24 h, where the resulting residual activity was 71.3% of that of the untreated enzyme [36]. On the other hand, work presented by Lanza et al. showed very low residual activity of exposed lipase from *Candida antarctica* at scCO_2_ conditions of 80 bar and 40 °C for 60 min. Their findings suggest that an increase in temperature and density led to an enhancement of the enzyme activity losses, while the decompression rates had a weak influence on enzyme inactivation [37].

The activities of enzymes in wheat flour were affected by the scCO_2_ process as well (Figure 2b). As the injected CO_2_ volume increased, the residual flour enzyme activity decreased. These results suggest that the rate of enzyme inactivation at the end of the scCO_2_ treatment depended significantly on pressure, treatment time, and the moisture content of the system. Studies of Primožič et al. demonstrated that an additional increase in pressure to 30 MPa causes a decrease in enzyme activity. The pressure elevation affected the activity of the native laccase, and, therefore, the structure of the enzyme could be modified, resulting in a decline in enzymatic activity [38]. Furthermore, the inactivation of enzymes in the wheat flour was not as successful as that of the native enzymes, as presented in Figure 2b. The residual activities of protease and lipase from the wheat flour after three hours of scCO_2_ treatment decreased by 25%, while the residual activities of α-amylase and peroxidase under the same conditions were almost unchanged. However, a decrease in residual activities of all four studied enzymes in wheat flour was observed at a higher pressure and at the same treatment time. A more significant decrease in α-amylase activity in wheat flour was observed by prolongation of the exposure time to 24 h of scCO_2_ processing (Figure 2b).

Various studies describe the effects of pressure and temperature under supercritical conditions with regard to the inactivation of enzymes, where ATPase and pectinase treated at conditions of 40 °C, 150 bar for 50 min acid phosphatase obtained the highest decrease in activity with a value of 78.4 (±3.9) [39]. Tedjo et al. presented the application of scCO_2_ treatment of peroxidase at 55 °C and 621 bar for 15 min, where peroxidase achieved approximately 65% inactivity [40]. Compared to that, in the present study, much higher inactivation of peroxidase was obtained at around half this pressure, that is, 300 bar, 35 °C, and three hours of exposure, where the residual activity of peroxidase was 12%.

Based on our results and previous assumptions about the inactivation of enzymes in association with amino acid residues of the enzyme, it can be emphasized that specific amino acid residues are important in the mechanism of inactivation with CO_2_. It is known that CO_2_ induces transient acidification during treatment, and, later, during degassing (CO_2_ expansion). In this regard, consistent with our results, some proteins could be subjected to conformational changes during acidification, including changes in surface hydrophobicity. For instance, Bednarski et al., reported that amino acid residue TRP retained its shape after exposure to scCO_2_ for thee hours. In our case, TRP is located in the alpha-amylase [41].

Table 2 shows the amino acid residues for each enzyme that we considered during our study. In addition to the four major amino acids in the active site of peroxidase, other minor residues have roles in the substrate interaction and catalytic cycle. All these amino acids show that peroxidases utilize many amino acids to accomplish a difficult reaction mechanism. Thus as previously demonstrated by Weder for lysozyme, during supercritical treatment with CO_2_, unfolding occurred, but, overall, no deleterious effect could be detected on amino acid composition [42]. Therefore, the scCO_2_ treatment targets primarily the enzyme proteins’ secondary structure (α-helix and β-pleated sheets). The enzyme inactivation mechanism most probably includes disruptive changes in the active site, unfavorable substrate desolvation, blockage of substrate access, and effects of transition state destabilization and restriction of conformational mobility.

The two-way ANOVA confirmed statistical differences between the enzyme types (*p* = 0.02) and enzyme inactivation in wheat flour (*p* < 0.01). There was no statistically significant interaction between the inactivation of enzymes in native form and enzymes in flour (F(3, 24) = 1.83, *p* = 0.17). Tukey multiple comparisons of means confirmed that enzyme inactivation was statistically different between enzyme groups (*p* = 0.03), and that α-amylase inactivation was statistically different (*p* < 0.05) from all other enzyme groups.

Overall, if we focus on the inactivation of enzymes in food, non-thermal treatments are certainly more desirable and attractive, with minimal or no impact on key nutritional and quality parameters [49]. Specifically, the native enzymes considered in our study are more sensitive to treatment with CO_2_ than the enzymes present in flour. The reason is that the scCO_2_ media do not inactivate enzymes in wheat flour directly, as the enzymes are located in an aleurone layer above the endosperm of the grain and are, thus, more difficult to access compared to native enzymes.

These findings indicate that the scCO_2_ is a promising technique for efficient inactivation of enzymes in flour, and, as far as we know, the use of supercritical CO_2_ for enzyme inactivation in flour has not yet been described in the literature.

### 3.4. Flour Particle Size Distribution

Further experiments were conducted to explain the effect of scCO_2_ exposure on flour particle size, since the experiments in this study are important for white wheat flour growers, millers, and the bakery industry, as this type of flour is still among the most highly consumed according to OECD.Stat.

**Figure 3:**
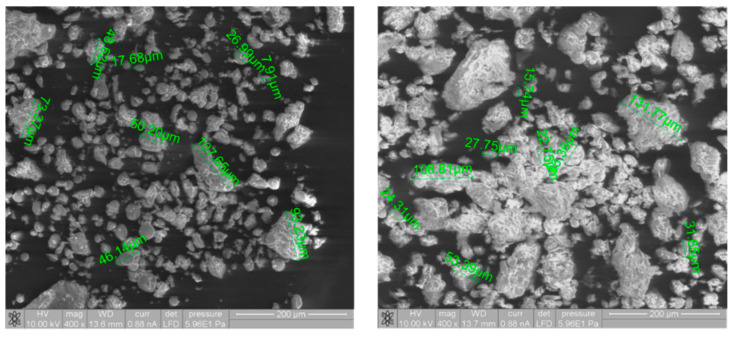
Environmental Scanning Electron Microscopy (ESEM) images of native white wheat flour (left) and white wheat flour after scCO_2_ treatment (right) at a magnification of 400×.

A laser diffraction particle size analyzer was used for the granulometric analysis of the wheat flour T500. A comparison of the particle sizes of untreated wheat flour and scCO_2_-treated wheat flour was made, where the average diameter of the untreated wheat flour granules was 60.6 µm and the average diameter of the scCO_2_-treated (300 bar, three hours) wheat flour granules was 70.7 µm. The increase in the average diameter of the wheat flour particle size can be attributed to the formation of aggregates, which is clearly evident when looking at Figure 3, showing the results of the ESEM analyses due to the presence of CO_2_ during exposure. The particle size measurements were repeated after a certain time (one week). The results demonstrated that the average particle size of wheat flour treated with scCO_2_ decreased to 61.2 µm, and after two weeks, it became equal to the initial value of the average particle size of untreated wheat flour (60.3 µm).

Regarding these analyses, we confirm that exposure to the scCO_2_ medium does not affect the morphological properties of the wheat flour, and does not result in any damage to the flour components, which are important in the further use of flour in the production of bakery products.

### 3.5. Environmental Scanning Electron Microscopy Studies

ESEM studies were used to determine the morphological characteristics of the starch grains. The microstructures of the flour samples were observed using ESEM (Figure 3), with irregularly shaped starch granules of different sizes being found. A comparison of the flour particles in the ESEM images showed that the scCO_2_-treated wheat flour particles appear to be larger in comparison to those of untreated wheat flour. After the scCO_2_ exposure significant particle size enlargement occurred, and microscopic observations observed spherical and ovoid granules of different sizes. Due to the influence of pressure and the addition of CO_2_ after exposure, some of the particles of flour aggregated, and this increased the average size of the scCO_2_ treated wheat flour. The most important observation with regard to the scCO_2_ treated wheat particles is that no mechanical damage on starch grains was caused due to the treatment. With ESEM studies, the effect of scCO_2_ on flour particles was observed. It has been proven that starch granules were not damaged by flour exposure to scCO_2_ and remains intact, which is a significant property for further use of flour in final bakery products.

### 3.6. CO_2_ Solubility in Flour

**Figure 4:**
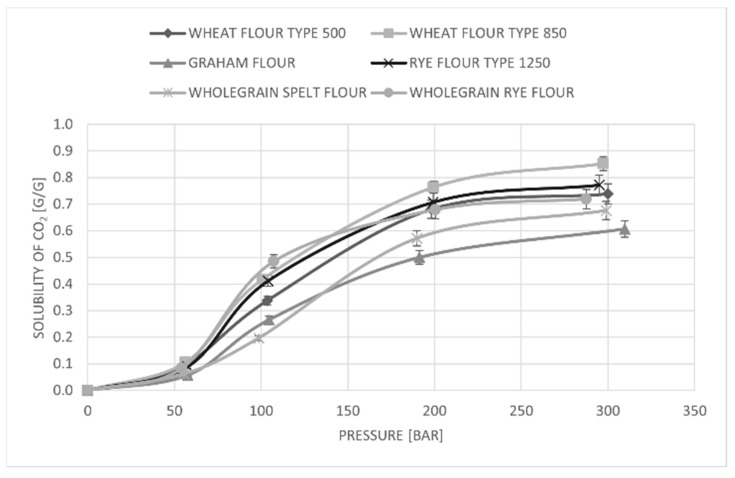
Comparison of CO_2_ solubility in different flour types at 35 °C.

The CO_2_ solubility in different flour types was determined with a magnetic suspension balance (MSB), where the temperature and pressure range considered for these experiments were the same as the conditions of the scCO_2_ treatment used for enzyme inactivation. The solubility of CO_2_ in different flour types was measured at 35 °C. The obtained results are presented in Figure 4, where it was observed that the solubility of CO_2_ increased with pressure. The solubility of CO_2_ at 300 bar was the highest in wheat flour type 850 and rye flour type 1250, with a value of 0.85 g/g. Afterwards, the solubility of CO_2_ was a little lower in wheat flour type 500, continued with wholegrain rye flour and wholegrain spelt flour. The lowest solubility of CO_2_ was found in Graham flour (0.6 g/g). In addition, when the flour is exposed to a dense gas, penetration of the gas molecules into the flour occurs and starts changing the density of the flour–gas mixture. The number of penetrating molecules rises when using gas with higher permeability and more porous material. CO_2_ is recognized as a highly permeable gas, which can increase the density of a sample [50].

An important finding regarding the solubility of CO_2_ in different flour types is that the solubility of CO_2_ goes hand-in-hand with the protein concentration in the flour. Table 1 shows that wheat flour 850 has a lower protein content than wheat flour 500 and, at the same time, has a higher solubility of CO_2_. This may be proof that a lower protein concentration is somehow connected with the higher solubility of CO_2_. The same observation was found for rye flour. Namely, rye flour type 1250 has a lower protein content (9.0 g/100 g flour) than wholegrain rye flour (13.0 g/100 g flour), and, at the same time, higher solubility of CO_2_. On the other hand, Graham flour had the highest protein value and the lowest solubility of CO_2_.

### 3.7. Fourier Transform Infrared Spectroscopy

**Figure 5:**
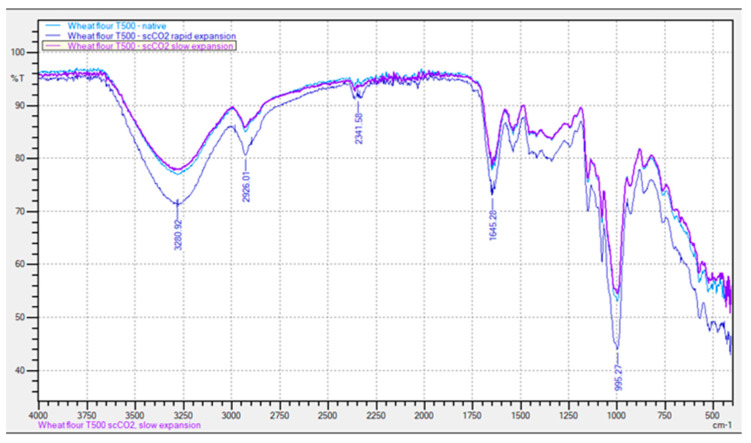
Comparison of the FTIR spectrum for non-treated and scCO_2_-treated wheat flour T500 with slow and rapid expansion of CO_2_.

According to the above investigations, the treatment of wheat flour with scCO_2_ medium had a significant influence on enzymatic activity. In further research, the influence of scCO_2_ treatment on the primary structure and conformation of the non-treated wheat flour and the scCO_2_-treated wheat flour were investigated by employing an FTIR instrument. The results shown in Figure 5 indicate that no significant changes in the infrared spectrum of the treated wheat flour in scCO_2_ medium were observed in comparison with the untreated wheat flour. Nevertheless, the infrared spectrum of wheat flour T500 after scCO_2_ treatment shows stronger adsorption intensities for all characteristic peaks between the samples. The absorption spectrum of wheat flour shows a strong peak for water with a stretching vibration of O-H bonds at 3260 cm^−1^, which represents the presence of moisture in the flour [51]. Furthermore, peaks in the range from 3000 cm^−1^ to 2800 cm^−1^ correspond to stretching vibrations of the C-H bond, which could be attributed to the presence of polysaccharides, unsaturated lipids, and carbohydrates.

To evaluate the effects of scCO_2_ treatment on the nutritional value of wheat flour, it was important to focus on the protein regions. According to the results from the FTIR analysis, the intensity of the absorption of the amide I band (centered at 1645 cm^−1^) increased due to the scCO_2_ treatment. Moreover, the characteristically strong peak at 1645 cm^−1^ is also attributed to the stretching vibrations of N-H, which presents mainly the bands of protein, and can be attributed to amide I and amine II [52]. A similar pattern was determined by Guzmán-Ortiz et al. for soybean (*Glycine max* L.) seeds and white corn (Zea mays), where high temperature extrusion caused changes in the secondary, tertiary, and quaternary protein structures [53].

### 3.8. Changes in Wheat Lipid Content during the scCO_2_ Treatment

ScCO_2_ may have an influence on the lipid content in the sample. Therefore, the total lipid content in white wheat flour was determined after exposure to the scCO_2_. Moreover, the presence of lipids is important with regard to bakery product production. The percentage of lipids obtained by extraction from untreated white wheat flour was 1.3%, while the percentage of lipids in white wheat flour treated with scCO_2_ at 300 bar and 24 h was 0.7%. The total lipid content differed significantly between the untreated and scCO_2_-treated white wheat flour. Therefore, based on the obtained results, the percentage of lipid loss in white wheat flour after scCO_2_ treatment was recalculated to a 46.15% loss. This is due to the lipids being soluble in CO_2,_ and being released from the high-pressure batch reactor, together with the CO_2_ gas. A similar trend was noted by Shin et al., who reported that scCO_2_ extraction (334.4 bar, 60 °C for 150 min) reduced the fat content of soy flour significantly from 19.5% to 7.1% [54]. Many factors influence flour’s shelf life or the length of time it lasts before beginning to spoil. Most flours stay fresh for several months at room temperature, usually long past the expiration date. However, the specific shelf life depends on the type of flour, its ingredients, and method of storage. ScCO_2_ technology holds the key to prolonging a product’s shelf life while reducing enzyme activity. By inactivating certain unwanted enzymes, it is assumed that this affects extending the shelf life of flour, as adverse reactions that affect the quality of the product occur more slowly. During the research, we performed experiments where we put the scCO_2_-treated flour and untreated flour for storage in the warehouse in a bakery, with which we cooperated during this research. Thus, we took a flour sample every few months and determined the activity of the enzymes in the stored flour. We also found that in scCO_2_-treated flour, bugs did not develop, as we know that scCO_2_ also affects the inactivation of microorganisms in food. Moreover, lipids play an essential role in food products’ shelf life and physical and sensory properties, as they present different structures, which affect their behavior, stability, plasticity, and texture. Certainly, due to the lower lipid content, the energy value of scCO_2_-treated wheat flour is lower than that of untreated white wheat flour, and, thus, pre-treated flour has a longer shelf life.

### 3.9. Sensory Evaluation of Baked Bread

**Figure 6:**
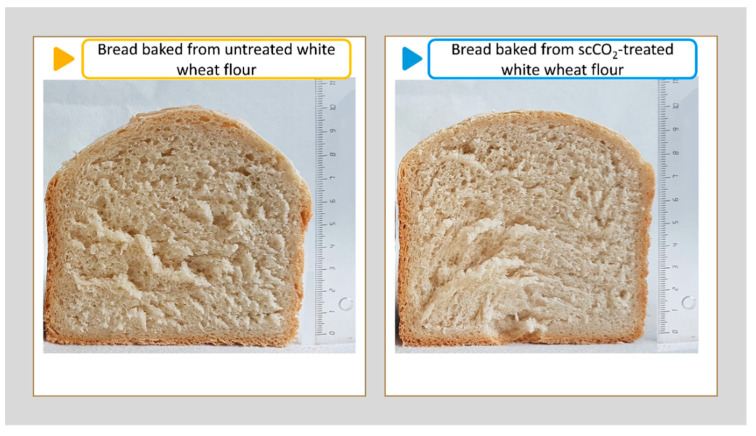
Samples of bread products (left–untreated white wheat flour; right–scCO_2_-treated white wheat flour).

Baked loaves of bread from untreated and scCO_2_-treated white wheat flour were evaluated for a sensory analysis. The aroma, taste, and texture were evaluated during the sensory analysis, which was performed immediately after the cooling process in the process of baking the bread. The final baked products from the untreated and scCO_2_-treated wheat flour were slightly different visually, as shown in the representative samples in Figure 6. The average height of the baked bread was 10.3 cm from the untreated white wheat flour, while the average height of the bread baked with the scCO_2_-treated wheat flour was 9.7 cm. However, a slightly lower bread was expected with the scCO_2_-treated white wheat flour, as the enzyme activity in the scCO_2_-treated white wheat flour was reduced due to the impact of the scCO_2_. Measurements of the specific volume of the bread were also carried out, and it was found that the bread from the untreated white wheat flour has a specific volume 2.77 ± 0.15 cm^3^/g, which is slightly higher than bread baked from the scCO_2_-treated white wheat flour with a specific volume 2.61 ± 0.18 cm^3^/g. Besides the effect of scCO_2_ on the baking properties of white wheat flour, the reduced activity of α-amylase affected the baking properties significantly. Certainly, it has been proven that it is possible to prepare the final product with scCO_2_-treated flour successfully. Bread baked under the same conditions is only slightly lower, and retains all the same properties as bread baked with untreated white wheat flour. Furthermore, the air spaces in the bread are comparable, which is a confirmation of a successful fermentation process.

## 4. Conclusions

The scCO_2_ process presents a sustainable and green method for enzymes’ inactivation in flour, which was presented for the first time in this study. The protein concentration was determined for six different types of flour, and the activities were determined of the enzymes α-amylase, protease, peroxidase, and lipase in white wheat flour. In the present study, for the first time, attempts were made to investigate the factors leading to enzyme inactivation by the interaction between proteins and scCO_2_ in a high-pressure batch reactor, to assess the impact of scCO_2_ conditions on flour quality, and, at the same time, to prolonged flour storage. As expected, it was found that scCO_2_ treatment causes the inactivation of both native enzymes and enzymes in wheat flour under certain conditions. It was found that the scCO_2_ treatment does not change or affect the quality of the treated flour negatively, which was confirmed using different analytical methods. In particular, it was observed on the ESEM images that, due to the influence of pressure and the addition of CO_2_ after exposure of wheat flour to scCO_2_, the granules bound together and increased in volume. However, after a certain time, the shape and size returned to their original states, which are equal to those of the non-treated sample. Moreover, an important finding that has not been reported before in the literature is that the solubility of CO_2_ in various flour types goes hand-in-hand with the protein content of the flour sample. In this study, the potential was also evaluated of scCO_2_ treated white wheat flour as a raw material for bakery products, and the development of a low-fat white wheat flour. One of the most useful findings is the wheat flour’s lower lipid content after scCO_2_ treatment. The energy value of scCO_2_ treated wheat flour is less than that of untreated white wheat flour, and, thus, pre-treated flour has a longer shelf life.

## Figures and Tables

**Table 1:** Characteristics of different flour types.

Flour Type	Moisture Content (%)	Fat Concentration (g/100 g Flour)	Protein Concentration (g/100 g Flour)
WHEAT FLOUR TYPE 500	13.1 ± 0.3	1.3 ± 0.5	12.0 ± 0.4
WHEAT FLOUR TYPE 850	13.3 ± 0.4	1.5 ± 0.6	11.0 ± 0.6
RYE FLOUR TYPE 1250	10.7 ± 0.3	1.3 ± 0.3	9.0 ± 0.3
WHOLEGRAIN RYE FLOUR	11.5 ± 0.2	1.7 ± 0.7	13.0 ± 0.5
GRAHAM FLOUR	11.0 ± 0.4	1.9 ± 0.5	13.4 ± 0.4

**Table 2:** Different amino acid residues by the enzyme.

Enzyme	Amino Acid Residues	Reference
α-amylase	Arg74, Trpl88, Tyrl90, Glu84	[43,44]
peroxidase	His170, Asp247, His42, Arg38, Phe41	[45]
lipase	Cys181, Ser152	[46,47]
protease	Asp30, His68, Ser255, Tyr195	[48]

## Data Availability

The data presented in this study are available on request from the corresponding author.
